# Supervised and Semisupervised Manifold Embedded Knowledge Transfer in Motor Imagery-Based BCI

**DOI:** 10.1155/2022/1603104

**Published:** 2022-10-17

**Authors:** Yilu Xu, Hua Yin, Wenlong Yi, Xin Huang, Wenjuan Jian, Canhua Wang, Ronghua Hu

**Affiliations:** ^1^School of Software, Jiangxi Agricultural University, Nanchang 330045, China; ^2^Software College, Jiangxi Normal University, Nanchang 330027, China; ^3^School of Information Engineering, Nanchang University, Nanchang 330031, China; ^4^School of Computer, Jiangxi University of Chinese Medicine, Nanchang 330004, China; ^5^School of Advanced Manufacturing, Nanchang University, Nanchang 330031, China

## Abstract

A long calibration procedure limits the use in practice for a motor imagery (MI)-based brain-computer interface (BCI) system. To tackle this problem, we consider supervised and semisupervised transfer learning. However, it is a challenge for them to cope with high intersession/subject variability in the MI electroencephalographic (EEG) signals. Based on the framework of unsupervised manifold embedded knowledge transfer (MEKT), we propose a supervised MEKT algorithm (sMEKT) and a semisupervised MEKT algorithm (ssMEKT), respectively. sMEKT only has limited labelled samples from a target subject and abundant labelled samples from multiple source subjects. Compared to sMEKT, ssMEKT adds comparably more unlabelled samples from the target subject. After performing Riemannian alignment (RA) and tangent space mapping (TSM), both sMEKT and ssMEKT execute domain adaptation to shorten the differences among subjects. During domain adaptation, to make use of the available samples, two algorithms preserve the source domain discriminability, and ssMEKT preserves the geometric structure embedded in the labelled and unlabelled target domains. Moreover, to obtain a subject-specific classifier, sMEKT minimizes the joint probability distribution shift between the labelled target and source domains, whereas ssMEKT performs the joint probability distribution shift minimization between the unlabelled target domain and all labelled domains. Experimental results on two publicly available MI datasets demonstrate that our algorithms outperform the six competing algorithms, where the sizes of labelled and unlabelled target domains are variable. Especially for the target subjects with 10 labelled samples and 270/190 unlabelled samples, ssMEKT shows 5.27% and 2.69% increase in average accuracy on the two abovementioned datasets compared to the previous best semisupervised transfer learning algorithm (RA-regularized common spatial patterns-weighted adaptation regularization, RA-RCSP-wAR), respectively. Therefore, our algorithms can effectively reduce the need of labelled samples for the target subject, which is of importance for the MI-based BCI application.

## 1. Introduction

A brain-computer interface (BCI) system can build a direct communication pathway for a subject between his brain and an electrical device without the participation of his peripheral nerves and muscles [1, 2]. Due to safety and convenience, noninvasive electroencephalogram (EEG)-based BCIs have attracted great attention for decades [3]. Diverse EEG paradigms have been widely studied, such as motor imagery (MI), event-related potentials (ERP), steady-state visual evoked potentials (SSVEP), emotion state recognition, and driver drowsiness estimation [4–10].

In this paper, we focus on MI-based BCI, where people with severe neuromuscular disabilities can recover their neurological disorders by spontaneously performing imagined movements of the feet, hands, or tongue without external stimuli [8]. Furthermore, MI-based BCI gives able-bodied people a novel way to control an electrical equipment [2]. Therefore, it is meaningful to study MI-based BCI.

However, EEG data analysis is challenging due to low signal-to-noise ratio and high artifacts [11, 12]. Moreover, a long calibration procedure hinders the development of MI-based BCI. Each subject usually spends a tedious calibration time in training a subject-specific classifier before performing the real-time MI tasks. Since the MI EEG signals are evoked by spontaneous movement imagination without external stimuli, they are of high intersession/subject variability. Thus, it is difficult to build a generic classifier that fits all sessions/subjects. Instead, it is realistic to train a subject-specific classifier that usually requires sufficient labelled data from the subject. Nevertheless, a long calibration procedure may unfortunately lead to high intersession differences and user frustration.

To cope with this problem, it is crucial for a target subject to reduce the need of amounts of labelled samples and effectively utilize the available samples. Rapid progress in machine learning motivates a variety of studies on how to make full use of the available samples [13–18]. EEG dataset reduction can reduce the feature dimensionality of the available EEG signals and improve the system learning speed. However, it cannot reduce the calibration time for the target subject [19, 20]. Likewise, different polynomials-based recurrence algorithms are promising techniques for signal processing due to their special capabilities in feature extraction. Nevertheless, they also just shorten the computational cost instead of calibration time [21–23]. Deep learning has been widely used in computer vision, natural language processing, and physiological signal analysis [24–28]. Nevertheless, deep learning needs lots of labelled samples from the target subject to show its superiority. The artificial data generation method can generate numerous artificial labelled data by recombining the few original labelled data in the time and frequency domains [29]. However, this method highly relies on the quantity and quality of the labelled samples. Transfer learning is a popular machine learning technique, which usually transfers labelled samples from different source sessions/subjects for a new target session/subject with no or few labelled samples [30]. Semisupervised learning can use the limited labelled set and comparably large unlabelled set from the same subject simultaneously [31]. Therefore, we pay more attention to transfer learning and its combination with semisupervised learning.

In general, transfer learning can be divided into three categories: supervised transfer learning, unsupervised transfer learning, and semisupervised transfer learning, depending on whether the samples from the target domain are all labelled, all unlabelled, or partially labelled and unlabelled. It is noted that all samples from the source domains are labelled no matter whether transfer learning is supervised, unsupervised, or semisupervised. Here, a domain means a subject or a session. Then, a labelled domain consists of the labelled samples from a subject/session, while an unlabelled domain includes the unlabelled samples from a subject/session. To our best knowledge, most studies focus on the supervised transfer learning since it can effectively use the discriminative labelled samples from the target domain to select and adjust the labelled samples from the source domains. In fact, it is of great importance for unsupervised and semisupervised transfer learning algorithms to explore the geometric information embedded in the amounts of unlabelled samples from the target domain. As an unsupervised transfer learning algorithm, manifold embedded knowledge transfer (MEKT) performed Riemannian alignment (RA), tangent space mapping (TSM), and domain adaptation to continually minimize the differences among different domains in ERP-based and MI-based BCIs [32]. In our opinion, it is difficult to apply unsupervised transfer learning to MI-based BCI due to the existence of BCI illiteracy. It is better to collect initial labelled samples from the target domain.

Therefore, inspired by MEKT, we develop a supervised MEKT algorithm (sMEKT) and a semisupervised MEKT algorithm (ssMEKT) to explore more possibilities of MEKT in all cases and improve the efficiency of transfer learning by using all available samples. The main contributions of our work can be summarized as follows:

1. We extend MEKT in the supervised and semisupervised versions to further testify the effectiveness of transfer learning on the Riemannian manifold and its tangent space for MI-based BCI.
2. We propose a supervised transfer learning algorithm (sMEKT) which transfers a large labelled source domain to a small labelled target domain by performing domain adaptation between the labelled domains.
3. We present a semisupervised transfer learning algorithm (ssMEKT) which transfers a large labelled source domain into the labelled and unlabelled target domains by performing domain adaptation between the unlabelled target domain and all labelled domains.

The rest of this paper is structured as follows. In Section 2, we introduce the related work on supervised, unsupervised, and semisupervised transfer learning. In Section 3, we present the applied MI datasets and the detailed methods of EEG processing, including preprocessing, RA, TSM, all MEKT based algorithms, and a shrinkage linear discriminant analysis (sLDA) classifier. The results of classification accuracy and computation time are shown in Section 4. The experimental results are discussed in Section 5. Finally, our conclusions are drawn in Section 6.

## 2. Related Work

Besides MI, the other EEG paradigms mentioned above, such as SSVEP and ERP, also use transfer learning to reduce or suppress the calibration time. Thus, different EEG-based BCIs can learn from each other in terms of transfer learning.

Most transfer learning algorithms are supervised, which are designed to address the shortage of labelled samples from the target domain. In MI-based BCI, the common spatial patterns (CSP) method is a classical feature extraction algorithm for only one subject. However, it performs poorly in the small labelled set scenario [33, 34]. Originated from CSP, regularized CSP (RCSP) calculated the regularized average spatial covariance matrix for each class by giving the labelled samples from the source and target domains' different regularization parameters [35]. Based on the framework of RCSP, different distance metrics, such as Frobenius norm and cosine distance, were used to measure the similarity between the labelled source and target domains [36, 37]. Combined CSP (CCSP) simply concatenated the labelled samples from the target domain and multiple source domains with equal weight to compute the spatial filters [38]. A dynamic time warping RCSP (DTW-RCSP) method performed domain adaptation by aligning the labelled source domains to the labelled target domain from the same class using an optimal warping path [39]. These RCSP algorithms inherited the advantage of the CSP in MI-based BCI. However, the regularization parameters, which were used to evaluate the differences between the labelled source and target domains, were often manually set or were obtained by means of cross-validation. Recently, since affine transformation can make the covariance matrices of EEG data from different domains close, RA-based supervised transfer learning algorithms have received widespread attention in different EEG-based BCIs [40–43]. Zanini first performed RA for each domain using the Riemannian mean of its resting trials as the reference matrix and then concatenated all aligned matrices from the labelled domains to train a minimum distance to mean (MDM) classifier based on Riemannian Gaussian distributions [44]. A Riemannian procrustes analysis (RPA) algorithm executed the following transformations (translation, scaling, and rotations) for the covariance matrices from the source and target domains to shorten their differences and then constructed an MDM classifier using the transformed matrices from the labelled domains [45]. Due to good performance of RA, our proposed sMEKT also belongs to the RA-based supervised transfer learning algorithms.

Many unsupervised transfer learning algorithms utilize the unlabelled samples from the target domain, as well as abundant labelled samples from the source domains to realize the zero-training for the target domain. In ERP-based BCI, Waytowich presented an unsupervised transfer learning method, where independent models were first trained using labelled samples from different source subjects, then the classification decisions of independent models were combined to classify unlabelled samples from the target subject, and finally each model's decision was weighted based on the inferred accuracy of direct classification [46]. Such method did not utilize the inherent information embedded in the unlabelled samples. In MI-based BCI, Xu proposed an unsupervised cross-dataset transfer learning, where EEGNet and ShallowConvNet were trained with the labelled source dataset, then an unsupervised domain adaptation was performed between the labelled source dataset and the unlabelled target dataset, and finally the pretrained model was validated on the unlabelled target dataset [47]. However, this algorithm did not realize the domain adaptation between the subjects. To minimize the differences among subjects, in ERP-based and MI-based BCIs, MEKT first executed RA for the covariance matrices from different subjects, then extracted the tangent feature vectors in the TSM module, and finally performed joint probability distribution shift minimization, labelled source domain discriminability preservation, and unlabelled target domain locality preservation [32]. Our proposed algorithms are based on the framework of MEKT, since MEKT can not only shorten the differences between domains but also preserve the characteristics of the labelled source domain and unlabelled target domain as much as possible.

Theoretically, semisupervised transfer learning can provide more information than unsupervised transfer learning due to the existence of a labelled target domain. To achieve epileptic seizure classification from EEG signals, Jiang integrated transfer learning, semisupervised learning, and a Takagi-Sugeno-Kang fuzzy system [48]. This method achieved good performance at the cost of the computation time. In ERP-based BCI, Wu proposed online and offline weighted adaptation regularization (wAR) algorithms which performed domain adaptation between the labelled source domain and the entire target domain by integrating the loss function minimization, structural risk minimization, marginal conditional probability distribution adaptation, and conditional probability distribution adaptation [49]. Although wAR is a semisupervised transfer learning algorithm, its architect is similar to that of unsupervised MEKT.

In summarize, these approaches mentioned above inspired the design of our supervised and semisupervised transfer learning algorithms in MI-based BCI.

## 3. Materials and Methods

### 3.1. Description of Datasets

Two publicly available MI datasets were used to assess the effectiveness of our proposed transfer learning algorithms. More details about these datasets are described as follows:

1. Dataset 1(BCI Competition III dataset IVa): this dataset contained 118-channel EEG signals from five healthy subjects (aa, al, av, aw, and ay). A visual cue on a computer screen was displayed for 3.5 s, during which the subject was instructed to perform one of the following MI tasks: left hand, right hand, and right foot. Only EEG signals evoked by the right hand and right foot MI tasks were provided for competition. A total of 140 trials per MI task were available for each subject. The EEG signals were band-pass filtered between 0.05 and 200 Hz and downsampled from 1000 Hz to 100 Hz [50].
2. Dataset 2 (BCI Competition IV dataset 1): this dataset consisted of the calibration data and evaluation data from seven healthy subjects (a, b, c, d, e, f, and g). Only calibration data was used for our experiments because of complete marker information. Two classes of MI tasks could be chosen from the three classes (left hand, right hand, and foot) by each subject. Each subject was shown a visual cue for a period of 4 s and performed the cued MI task. 100 trials per MI task were collected for each subject. 59-channel EEG signals were recorded at a sampling rate of 100 Hz and band-pass filtered between 0.05 and 200 Hz [51].

### 3.2. The Frameworks of Different MEKT Based Algorithms

**Figure 1:**
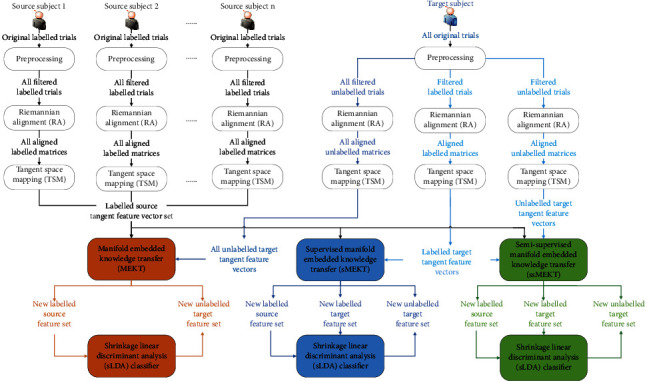
The frameworks of MEKT [32], sMEKT, and ssMEKT.

Our proposed supervised MEKT (sMEKT) and semisupervised MEKT (ssMEKT) are based on the unsupervised MEKT. To better understand them, the frameworks of MEKT, sMEKT, and ssMEKT are shown in Figure 1.

The detailed steps of the different algorithms are outlined below:

1. Preprocess the original EEG trials from different subjects. Note that the original EEG trials from all source subjects are labelled and used for all algorithms. For MEKT, all original EEG trials from the target subject are unlabelled. However, for sMEKT and ssMEKT, they are partitioned into the labelled and unlabelled EEG trials. The labelled ones are used for sMEKT and ssMEKT, whereas the unlabelled ones are only used for ssMEKT.
2. Convert the filtered labelled/unlabelled EEG trials from each subject into the corresponding labelled/unlabelled covariance matrices. Then, we perform RA for these covariance matrices using their Riemannian mean as the reference matrix to obtain the corresponding aligned matrices in different MEKT based algorithms.
3. In the TSM module, the aligned matrices are transformed into the Euclidean tangent feature vectors. The tangent feature vectors from different source subjects are concatenated into the labelled source tangent feature vector set, which is inputted into ssMEKT, MEKT, and sMEKT along with all, unlabelled, and labelled target tangent feature vectors, respectively. It is noted that the target tangent feature vectors are the feature vectors from the target subject.
4. All MEKT based algorithms utilize the available tangent feature vector sets to yield different projection matrices which can be used to generate new lower dimensional feature sets.
5. All new labelled feature sets from different subjects are fed into the sLDA classifier [52] to train a subject-specific model which is then used to classify the new unlabelled target feature set.

Next, we introduce the above procedures in detail.

### 3.3. Preprocessing of EEG Data

For dataset 1, all original EEG trials from each subject were band-pass filtered between 8 and 30 Hz using a fifth-order Butterworth filter. Then, the filtered EEG trials were extracted from the time interval between 0.5 and 2.5 s after the visual cue signalling the start of imagery.

For dataset 2, all original EEG trials from each subject were spectrally filtered by a fiftieth order finite impulse response filter with cut-off frequencies of 8 and 30 Hz and temporally segmented from 0.5 to 3.5 s after the visual cue onset.

**Figure 2:**
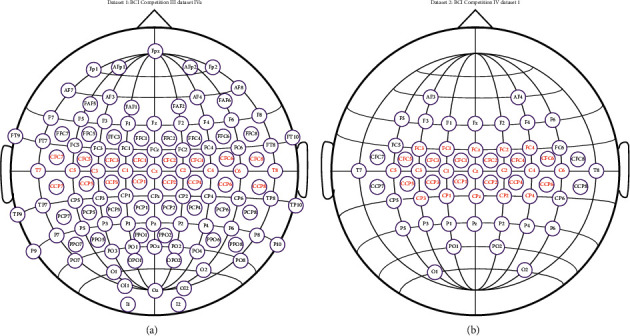
The selected channels for two datasets. (a) Dataset 1. (b) Dataset 2.

We only used channels in the central area of the brain where the sensorimotor rhythms (SMR) of MI are active. 25 and 29 channels were selected separately for dataset 1 and dataset 2. In Figure 2, the selected channels for the two datasets are marked in red.

### 3.4. Riemannian Alignment

Although there are high intersubject variances in MI-based BCI, RA can make the marginal distributions of EEG trials from different subjects closer.

Let *X*_*i*_ ∈ *ℝ*^*n*×*t*^ be the *i*th filtered trial from a subject, where *n* is the number of channels and *t* is the number of sample points of the selected time window. The spatial covariance matrix of *X*_*i*_ can be defined as [40]

(1) $$\begin{array}{c} C_{i}=\frac{X_{i}X_{i}^{T}}{t-1}\text{.} \end{array}$$

Since the covariance matrices belong to a smooth Riemannian manifold of symmetric positive definite (SPD) matrices, they can be viewed as points on the manifold [44]. To show the benefits of RA, we first present some basic concepts of Riemannian geometry.

#### 3.4.1. The Riemannian Distance

Suppose that *C*_*i*_ and *C*_*j*_ are the points of the Riemannian manifold. The Riemannian distance *δ*(*C*_*i*_, *C*_*j*_) between these two points can be defined as the length of the shortest curve (named geodesic) connecting them [53]:

(2) $$\begin{array}{c} \delta \left(C_{i},C_{j}\right)={\left\|\log\;\left(C_{i}^{-1}C_{j}\right)\right\|}_{F}={\left[\overset{n}{\underset{k=1}{\sum }}\log^{2}\lambda_{k}\right]}^{1/2}\text{,} \end{array}$$

with the Frobenius norm ‖∙‖_*F*_ and the eigenvalues ({*λ*_*k*_}_*k*=1_^*n*^) of *C*_*i*_^−1^*C*_*j*_.

#### 3.4.2. The Riemannian Mean

The Riemannian mean is usually used as the statistical descriptor of a set of SPD matrices on the manifold. Given *N* SPD matrices, their Riemannian mean *M*_*R*_ is defined as below [44]:

(3) $$\begin{array}{c} M_{R}=\underset{M}{argmin}\overset{N}{\underset{k=1}{\sum }}\delta^{2}\left(C_{k},M\right)\text{.} \end{array}$$

For *N*=2, *M*_*R*_ is the middle point of a geodesic connecting the two points. However, *M*_*R*_ can be effectively calculated by an iterative procedure for *N* > 2 [40].

#### 3.4.3. Congruence Invariance

Congruence invariance is an important property about the Riemannian distance, which means that the distance between the two points remains invariant after affine transformation using an invertible matrix as below [45]:

(4) $$\begin{array}{c} \delta \left(C_{i},C_{j}\right)=\delta \left({W^{T}C}_{i}W,W^{T}C_{j}W\right)\forall W\in GL\left(n\right)\text{,} \end{array}$$

where *GL*(*n*) is the set of invertible symmetric *n* × *n* square matrices.

Based on these concepts, RA executes the affine transformation for *N* points on the manifold using their Riemannian mean as the reference matrix. Then, the aligned matrix of *C*_*i*_ is [44]

(5) $$\begin{array}{c} \hat{C_{i}}=M_{R}^{\left(-1/2\right)}C_{i}M_{R}^{\left(-1/2\right)}\text{.} \end{array}$$

We perform RA for all covariance matrices from each domain using their own Riemannian mean as the reference matrix. Then, all aligned matrices from each domain are centred at the identity matrix. This property can be testified by the following [32]:

(6) $$\begin{array}{c} M\left(M_{R}^{-1/2}C_{1}M_{R}^{-1/2},{M_{R}^{-1/2}C}_{2}M_{R}^{-1/2},\cdots ,M_{R}^{-1/2}C_{N}M_{R}^{-1/2}\right)\\ =M_{R}^{-1/2}M\left(C_{1},C_{2},\cdots ,C_{N}\right)M_{R}^{-1/2}=M_{R}^{-1/2}M_{R}M_{R}^{-1/2}=I\text{,} \end{array}$$

where *ℳ*(*C*_1_, *C*_2_, ⋯, *C*_*N*_) is the Riemannian mean operation and *I* is an identity matrix.

Thus, RA can make the aligned matrices from different domains comparable and preliminarily shorten their differences.

Additionally, as in [40], the filtered trial *X*_*i*_ can be spatially whitened by performing RA as follows:

(7) $$\begin{array}{c} {\hat{X}}_{i}=M_{R}^{\left(-1/2\right)}X_{i}\text{.} \end{array}$$

### 3.5. Tangent Space Mapping

Most traditional classifiers, such as linear discriminant analysis (LDA) and support vector machine (SVM), are designed for the Euclidean space, instead of the Riemannian space. To inherit the advantage of RA, we transform the aligned matrices into the Euclidean tangent feature vectors.

**Figure 3:**
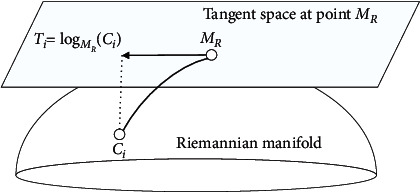
A Riemannian manifold and its tangent space (this figure was adopted from Barachant et al. [40]).

The SPD matrices lie in a differentiable Riemannian manifold. Their derivatives at a reference point on the manifold compose a tangent space. As mentioned in [54], the choice of Riemannian mean for the reference point leads to a tangent space locally approximate to the manifold.  Figure 3 shows a Riemannian manifold and its tangent space at a Riemannian mean point *M*_*R*_.

As shown in Figure 3, the logarithmic map projects *C*_*i*_ onto the tangent space at a Riemannian mean point *M*_*R*_ by [40]

(8) $$\begin{array}{c} T_{i}=\log_{M_{R}}\left(C_{i}\right)=M_{R}^{1/2}\log\;\left(M_{R}^{-1/2}C_{i}M_{R}^{-1/2}\right)M_{R}^{1/2}\text{.} \end{array}$$

As in (6), the identity matrix *I* is the Riemannian mean of all aligned matrices from each domain. Then, the logarithmic mapping of the aligned matrix ${\hat{C}}_{i}$ onto the normalized tangent space can be calculated as follows [55]:

(9) $$\begin{array}{c} {\hat{T}}_{i}=\log_{I}\left({\hat{C}}_{i}\right)=\log_{I}\;\left(M_{R}^{-1/2}C_{i}M_{R}^{-1/2}\right)=\log\;\left(M_{R}^{-1/2}C_{i}M_{R}^{-1/2}\right)=M_{R}^{-1/2}\log_{M_{R}}\left(C_{i}\right)M_{R}^{-1/2}\text{.} \end{array}$$

To obtain a minimal representation, we vectorize the above logarithmic mapping ${\hat{T}}_{i}$ by keeping its upper triangular part and applying unity weight for its diagonal elements and $\sqrt{2}$ weight for its nondiagonal elements [40]:

(10) $$\begin{array}{c} F_{i}=vect\left({\hat{T}}_{i}\right)=\left[{{\;\hat{T}}_{i}}_{1,1};{{\sqrt{2}\hat{T}}_{i}}_{1,2};{{\;\hat{T}}_{i}}_{2,2};{{\sqrt{2}\hat{T}}_{i}}_{1,3};{\sqrt{2}{\hat{T}}_{i}}_{2,3};{{\;\hat{T}}_{i}}_{3,3};\cdots ;{{\;\hat{T}}_{i}}_{n,n}\right]\text{,} \end{array}$$

where ${\hat{T}}_{i_{j,k}}\in {\hat{T}}_{i}$. Then, the aligned matrix ${\hat{C}}_{i}$ is transformed into the tangent feature vector *F*_*i*_.

### 3.6. Manifold Embedded Knowledge Transfer

The tangent feature vectors from different domains have similar marginal probability distributions inherited from the corresponding aligned matrices. However, their dimensionality *d*=*n* × (*n*+1)/2 is very high. To further reduce their differences and dimensionality, MEKT based algorithms aim to find optimal projection matrices for these tangent feature vectors.

As shown in Figure 1, all labelled tangent feature vectors from multiple source subjects are concatenated into a labelled source tangent feature vector set. For convenience, this set is called a labelled source domain *F*_*S*_={*F*_*S*,*i*_}_*i*=1_^*n*_*S*_^. Let *F*_*S*,*i*_ and *n*_*S*_ be the *i*th tangent feature vector and the size of *F*_*S*_, respectively. Likewise, all tangent feature vectors from the target subject can be called a target domain *F*_*T*_={*F*_*T*,*i*_}_*i*=1_^*n*_*T*_^. Assume *F*_*T*,*i*_ and *n*_*T*_ are the *i*th tangent feature vector and the size of *F*_*T*_, respectively. For MEKT, *F*_*T*_ is unlabelled.

Since MEKT transfers a labelled source domain to an unlabelled target domain, it is an unsupervised transfer learning algorithm.

MEKT seeks the optimal projection matrix *P*_*S*_ ∈ *ℝ*^*d*×*q*^ for *F*_*S*_ and the optimal projection matrix *P*_*T*_ ∈ *ℝ*^*d*×*p*^ for *F*_*T*_, which can not only make the lower dimensional features *P*_*S*_^T^*F*_*S*_ and *P*_*T*_^T^*F*_*T*_ close, but also preserve the labelled source domain discriminability and the unlabelled target domain locality. Note that *q* ≪ *d* is the dimensionality of a shared subspace for MEKT. MEKT sets *q* = 10. The following four properties are designed:

#### 3.6.1. Joint Probability Distribution Shift Minimization

The traditional maximum mean discrepancy (MMD) is usually used to shorten the marginal and conditional probability distribution discrepancies between different domains [56]. For simplicity, in MEKT, the joint probability MMD is proposed to measure and minimize the joint probability distribution shift between the source and target domains as follows [32]:

(11) $$\begin{array}{c} \min_{P_{S},P_{T}}D_{S,T}=\min_{P_{S},P_{T}}D\left(Q\left(F_{S},y_{S}\right),Q\left(F_{T},{\hat{y}}_{T}\right)\right)=\min_{P_{S},P_{T}}D\left(Q\left(F_{S}|y_{S}\right)Q\left(y_{S}\right),Q\left(F_{T}|{\hat{y}}_{T}\right)\left|Q\right.\left({\hat{y}}_{T}\right)\right)\approx \min_{P_{S},P_{T}}\left(\overset{2}{\underset{k=1}{\sum }}{\left\|\frac{1}{n_{S}}\overset{n_{S}^{k}}{\underset{i=1}{\sum }}P_{S}^{T}F_{S,i}^{k}-\frac{1}{n_{T}}\overset{n_{T}^{k}}{\underset{i=1}{\sum }}P_{T}^{T}F_{T,i}^{k}\right\|}_{F}^{2}\right)=\min_{P_{S},P_{T}}\;{\left\|N_{S}^{T}F_{S}^{T}P_{S}-N_{T}^{T}F_{T}^{T}P_{T}\right\|}_{F}^{2}\text{,} \end{array}$$

where *𝒟*(∙, ∙) is the joint probability distribution shift operation. *Q*(∙), *Q*(∙*|*∙), and *Q*(∙, ∙) are the marginal, conditional, and joint probability distributions, respectively. Assume *y*_*S*_ and ${\hat{y}}_{T}$ are the label vector of the labelled source domain and the pseudolabel vector of the unlabelled target domain, respectively. Let *n*_*S*_^*k*^ and *F*_*S*,*i*_^*k*^ be the size and the *i*th tangent feature vector of the labelled source domain belonging to the *k*th class, respectively. Likewise, *n*_*T*_^*k*^ and *F*_*T*,*i*_^*k*^ are the size and the *i*th tangent feature vector of the unlabelled target domain predicted to be the *k*th class, respectively. Here, only binary classification is considered. Then, *N*_*S*_=*Y*_*S*_/*n*_*S*_ and $N_{T}={\hat{Y}}_{T}/n_{T}$, where *Y*_*S*_ is the one-hot encoding matrix of *y*_*S*_, and ${\hat{Y}}_{T}$ is the one-hot encoding matrix of ${\hat{y}}_{T}$. The one-hot encoding matrix will be [1,0; 0,1; 0,1], if its corresponding true/pseudolabel vector is [class 1; class 2; class 2].

#### 3.6.2. Source Domain Discriminability Preservation

The source domain discriminability can be defined by the within-class and between-class scatter matrices. Thus, after projection, it can be maintained by [32]

(12) $$\begin{array}{c} \min_{P_{S}}tr\left(P_{S}^{T}S_{w}^{s}P_{S}\right),subject\;to:P_{S}^{T}S_{b}^{s}P_{S}=I\text{,} \end{array}$$

where tr(∙) is the trace computation, *S*_*w*_^*s*^=∑_*k*=1_^2^*F*_*S*_^*k*^*H*_*n*_*S*_^*k*^_*F*_*S*_^*k*^^T^ is the within-class scatter matrix of the labelled source domain, and $S_{b}^{s}=\sum_{k=1}^{2}n_{S}^{k}\left({\bar{m}}_{S}^{k}-{\bar{m}}_{S}\right){\left({\bar{m}}_{S}^{k}-{\bar{m}}_{S}\right)}^{T}$ is the between-class scatter matrix of the labelled source domain, in which *F*_*S*_^*k*^, *H*_*n*_*S*_^*k*^_=*I* − 1_*n*_*S*_^*k*^_/*n*_*S*_^*k*^, ${\bar{m}}_{S}^{k}$ represent the labelled source domain belonging to class *k*, its centring matrix, and its mean, respectively, and ${\bar{m}}_{S}$ is the mean of the labelled source domain [57]. Note that 1_*n*_*S*_^*k*^_ ∈ *ℝ*^*n*_*S*_^*k*^×*n*_*S*_^*k*^^ is an all-one matrix.

#### 3.6.3. Target Domain Locality Preservation

Although the target domain is unlabelled, its local manifold structure can be formulated by the graph Laplacian matrix. MEKT constructs the normalized Laplacian graph *L*=*I* − *D*^−1/2^*WD*^−1/2^ [32], where

(13) $$\begin{array}{c} W_{ij}=\left\{\begin{array}{cc} \exp\left(\frac{-{\left\|F_{T,i}-F_{T,j}\right\|}_{2}^{2}}{2\sigma^{2}}\right) & if\;F_{T,i}\in {Near}_{K}\left(F_{T,j}\right)\text{,}\\ 0\text{,} & otherwise \end{array}\right.\\ D_{ij}=\left\{\begin{array}{cc} \sum_{j=1}^{n_{T}}W_{ij}\text{,} & if\;i=j\text{,}\\ 0\text{,} & otherwise \end{array}\right. \end{array}$$

in which *σ* is a scaling parameter and Near_*K*_(*F*_*T*,*j*_) is a set of *K* nearest neighbours of *F*_*T*,*j*_ using the Euclidean metric. MEKT sets *σ*=1 and *K*=10.

To maintain the target domain locality after projection and remove the scaling effect, a graph regularization is minimized under the following constraints [32]:

(14) $$\begin{array}{c} \min_{P_{T}}\overset{n_{T}}{\underset{i,j=1}{\sum }}{\left\|P_{T}^{T}F_{T,i}-P_{T}^{T}F_{T,j}\right\|}_{2}^{2}W_{ij}=\min_{P_{T}}\text{ tr}\left(P_{T}^{T}F_{T}LF_{T}^{T}P_{T}\right),\\ subject\;to:P_{T}^{T}F_{T}H_{n_{T}}F_{T}^{T}P_{T}=I\text{,} \end{array}$$

where *H*_*n*_*T*__ = *I* − 1_*n*_*T*__/*n*_*T*_ is also a centring matrix.

#### 3.6.4. Parameter Transfer and Regularization

The following constraints are imposed on the projection matrices for better similarity and generalization performance [32]:

(15) $$\begin{array}{c} \min_{P_{S},P_{T}}\left({\left\|P_{S}-P_{T}\right\|}_{F}^{2}+{\left\|P_{T}\right\|}_{F}^{2}\right)\text{.} \end{array}$$

Then, the four properties above are integrated into an overall loss function of MEKT using different weights *α*, *β*, *γ*, and *θ* [32]:

(16) $$\begin{array}{c} \begin{array}{c} \min_{P_{S},P_{T}}\left({\alpha D}_{S,T}+\beta tr\left(P_{S}^{T}S_{w}^{s}P_{S}\right)+\gamma tr\left(P_{T}^{T}F_{T}LF_{T}^{T}P_{T}\right)+\theta \left({\left\|P_{S}-P_{T}\right\|}_{F}^{2}+{\left\|P_{T}\right\|}_{F}^{2}\right)\right)\\ subject\;to:P_{S}^{T}S_{b}^{s}P_{S}=I,P_{T}^{T}F_{T}H_{n_{T}}F_{T}^{T}P_{T}=I\text{,} \end{array}\text{,} \end{array}$$

where *α*, *β*, *γ*, and *θ* are manually set to be 1, 0.01, 0.1, and 20, respectively. For convenience, assume *P*=[*P*_*S*_;  *P*_*T*_] is an overall projection matrix (*P* ∈ *ℝ*^2 *d*×*q*^). The Lagrange function is designed as below [32]:

(17) $$\begin{array}{c} T=tr\left(P^{T}\left(\alpha A+\beta B+\gamma E+\theta G\right)P+\mu \left(I-P^{T}JP\right)\right)\text{,} \end{array}$$

where

(18) $$\begin{array}{c} A=\left[\begin{array}{cc} {F_{S}N_{S}N}_{S}^{T}F_{S}^{T} & -F_{S}N_{S}N_{T}^{T}F_{T}^{T}\\ -F_{T}N_{T}N_{S}^{T}F_{S}^{T} & F_{T}N_{T}N_{T}^{T}F_{T}^{T} \end{array}\right],B=\left[\begin{array}{cc} S_{w}^{s} & 0\\ 0 & 0 \end{array}\right],E=\left[\begin{array}{cc} 0 & 0\\ 0 & F_{T}LF_{T}^{T} \end{array}\right]\\ G=\left[\begin{array}{cc} I & -I\\ -I & 2I \end{array}\right],J=\left[\begin{array}{cc} S_{b}^{s} & 0\\ 0 & F_{T}H_{n_{T}}F_{T}^{T} \end{array}\right]\text{.} \end{array}$$

To obtain the optimal *P*, MEKT sets the derivative of *𝒯* to be 0 and then has

(19) $$\begin{array}{c} \left(\alpha A+\beta B+\gamma E+\theta G\right)P=\mu JP\text{.} \end{array}$$

Note that *μ*=10^−3^ is also a weight. After generalized eigen-decomposition, *P* is comprised of *q* trailing eigenvectors, where *q*=10 is the dimensionality of the new feature. Consequently, *P*_*S*_ and *P*_*T*_ can be obtained from *P*. For the matrix *A*, *N*_*T*_ relates to the pseudolabel vector ${\hat{y}}_{T}$. Since ${\hat{y}}_{T}$ is unknown initially, it is set to be an all-zero vector first. At the next iteration, ${\hat{y}}_{T}$ is updated using sLDA as the classifier. MEKT performs five iterations in total.

### 3.7. The Proposed Supervised Manifold Embedded Knowledge Transfer

Supervised manifold embedded knowledge transfer (sMEKT) is an extension of MEKT in the supervised version. For sMEKT, all tangent feature vectors from the target subject are divided into the labelled and unlabelled target domains. Only the labelled source and target domains are used to train a subject-specific classifier. The remaining unlabelled target domain is used to evaluate the performance of sMEKT.

To make full use of the available data, the following two problems should be taken into consideration for sMEKT:

1. How to choose the appropriate regularization terms and constraints in the formulation of sMEKT?
2. How to map the unlabelled target domain into the projected subspace since only the projection matrices for the labelled source and target domains are obtained for sMEKT?

This section presents the corresponding solutions to the above questions. Let *P*_*TL*_ be the projection matrix of the labelled target domain.

First, we exclude the target domain locality preservation because the limited labelled target domain does not effectively show the entire geometric structure of the target domain with the absence of the unlabelled target domain.

Secondly, we keep the source domain discriminability preservation as in (12) and do not add the labelled target domain discriminability preservation as

(20) $$\begin{array}{c} \min_{P_{TL}}tr\left(P_{TL}^{T}S_{w}^{TL}P_{TL}\right),subject\;to:P_{TL}^{T}S_{b}^{TL}P_{TL}=I\text{,} \end{array}$$

where *S*_*w*_^*TL*^ and *S*_*b*_^*TL*^ are the within-class scatter matrix and between-class scatter matrix of the labelled target domain, respectively. The reason is that sMEKT aims at transferring the labelled source domain for the target domain. Therefore, the source domain discriminability should be taken into consideration, instead of the target domain discriminability.

In addition, the crucial work of MEKT is to minimize the joint probability distribution shift between the source and target domains. In sMEKT, since we only have the labelled source and target domains, the joint probability distribution shift minimization is updated as below:

(21) $$\begin{array}{c} \min_{P_{S},P_{TL}}D_{S,TL}=\min_{P_{S},P_{TL}}D\left(Q\left(F_{S},y_{S}\right),Q\left(F_{TL},y_{TL}\right)\right)=\min_{P_{S},P_{TL}}D\left(Q\left(F_{S}\left|y_{S}\right.\right)Q\left(y_{S}\right),Q\left(F_{TL}\left|y_{TL}\right.\right)\left|Q\right.\left(y_{TL}\right)\right)\approx \min_{P_{S},P_{TL}}\left(\overset{2}{\underset{k=1}{\sum }}{\left\|\frac{1}{n_{S}}\overset{n_{S}^{k}}{\underset{i=1}{\sum }}P_{S}^{T}F_{S,i}^{k}-\frac{1}{n_{TL}}\overset{n_{TL}^{k}}{\underset{i=1}{\sum }}P_{TL}^{T}F_{TL,i}^{k}\right\|}_{F}^{2}\right)=\min_{P_{S},P_{TL}}\;{\left\|N_{S}^{T}F_{S}^{T}P_{S}-N_{TL}^{T}F_{TL}^{T}P_{TL}\right\|}_{F}^{2}\text{,} \end{array}$$

where *y*_*TL*_ and *n*_*TL*_ are the label vector and the size of the labelled target domain *F*_*TL*_, respectively, *n*_*TL*_^*k*^ and *F*_*TL*,*i*_^*k*^ are the size and the *i*th tangent feature vector of *F*_*TL*_ belonging to the *k*th class, and *N*_*TL*_=*Y*_*TL*_/*n*_*TL*_, in which *Y*_*TL*_ is the one-hot encoding matrix of *y*_*TL*_.

Finally, we remove the parameter transfer and regularization. In (15) and (16), MEKT pays more attention to minimization of the differences between *P*_*S*_ and *P*_*T*_ because it sets *θ*=20. In our opinion, this minimization can further shorten the gaps between the labelled source domain and the unlabelled target domain, which will benefit the classification of the latter. If we have the similar constraint, min_*P*_*S*_,*P*_*TL*__(‖*P*_*S*_ − *P*_*TL*_‖_*F*_^2^), and use the same weight on it, it may not play the same role as the constraint in (15) since the limited labelled target domain may not represent the remaining unlabelled target domain well. Calculating the optimal *θ* with cross-validation can make up this limitation. However, it will yield computational burden.

Thus, the overall loss function of sMEKT can be formulated by

(22) $$\begin{array}{c} \min_{P_{S},P_{TL}}\left({\alpha D}_{S,TL}+\beta tr\left(P_{S}^{T}S_{w}^{s}P_{S}\right)\right),subject\;to:P_{S}^{T}S_{b}^{s}P_{S}=I\text{.} \end{array}$$

Let *P*=[*P*_*S*_; *P*_*TL*_](*P* ∈ *ℝ*^2 *d*×*q*^). Then, the corresponding Lagrange function is

(23) $$\begin{array}{c} T=tr\left(P^{T}\left(\alpha A+\beta B\right)P+\mu \left(I-P^{T}JP\right)\right)\text{,} \end{array}$$

where

(24) $$\begin{array}{c} A=\left[\begin{array}{cc} {F_{S}N_{S}N}_{S}^{T}F_{S}^{T} & -F_{S}N_{S}N_{TL}^{T}F_{TL}^{T}\\ -F_{TL}N_{TL}N_{S}^{T}F_{S}^{T} & F_{TL}N_{TL}N_{TL}^{T}F_{TL}^{T} \end{array}\right],B=\left[\begin{array}{cc} S_{w}^{s} & 0\\ 0 & 0 \end{array}\right],J=\left[\begin{array}{cc} S_{b}^{s} & 0\\ 0 & 0 \end{array}\right]\text{.} \end{array}$$

Then, (23) can be solved by the same means as in (17). Since *Y*_*TL*_ in *N*_*TL*_ is the one-hot encoding matrix of the label vector *y*_*TL*_, rather than that of the pseudolabel vector, we can obtain the optimal *P* without multiple iterations.

As for the second question presented above, we assume that the labelled and unlabelled target domains have similar joint probability distributions. Moreover, we minimize the joint probability distribution shift between the labelled source and target domains in (21). Thus, we define the average of the projection matrices of the labelled source and target domains as the projection matrix of unlabelled target domain:

(25) $$\begin{array}{c} P_{TU}=\frac{\left(P_{S}+P_{TL}\right)}{2}\text{.} \end{array}$$

Therefore, the new unlabelled target feature set is *P*_*TU*_^T^*F*_*TU*_, where *F*_*TU*_ is the unlabelled target domain.

### 3.8. The Proposed Semisupervised Manifold Embedded Knowledge Transfer

Semisupervised manifold embedded knowledge transfer (ssMEKT) utilizes the labelled source and target domains, as well as the unlabelled target domain. We construct the following regularization terms and constraints for ssMEKT.

First, we keep the target domain locality preservation because of the existence of the labelled and unlabelled target domains. Let *F*_*T*_=*F*_*TL*_ ∪ *F*_*TU*_. It is noted that *F*_*TL*_ and *F*_*TU*_ are separately obtained after performing RA and TSM for their original domains. Then, we can minimize the graph regularization using all data from the target domain as in Section 3.6. Accordingly, we retain the projection matrix for the entire target domain, denoted as *P*_*T*_.

Then, like sMEKT, the source domain discriminability preservation is considered.

Additionally, to benefit the classification of the unlabelled target domain, we reduce the joint probability distribution discrepancies between the labelled and unlabelled domains, instead of those between the source and target domains. Actually, MEKT also minimizes the joint probability distribution shift between the labelled and unlabelled domains since the source domain is labelled and the target domain is unlabelled. For ssMEKT, the joint probability distribution shift minimization can be rewritten as

(26) $$\begin{array}{c} \min_{P_{S},P_{T},P_{TL}}D_{L,U}=\min_{P_{S},P_{T},P_{TL}}D\left(Q\left(F_{S}\cup F_{TL},y_{S}\cup y_{TL}\right),Q\left(F_{TU},{\hat{y}}_{TU}\right)\right)=\min_{P_{S},P_{T},P_{TL}}D\left(Q\left(F_{S}\cup F_{TL}|y_{S}\cup y_{TL}\right)Q\left(y_{S}\cup y_{TL}\right),Q\left(F_{TU}|{\hat{y}}_{TU}\right)|Q\left({\hat{y}}_{TU}\right)\right)\approx \min_{P_{S},P_{T},P_{TL}}\left(\overset{2}{\underset{k=1}{\sum }}{\left\|\frac{1}{n_{S}}\overset{n_{S}^{k}}{\underset{i=1}{\sum }}P_{S}^{T}F_{S,i}^{k}+\frac{1}{n_{TL}}\overset{n_{TL}^{k}}{\underset{i=1}{\sum }}P_{TL}^{T}F_{TL,i}^{k}-\frac{1}{n_{TU}}\overset{n_{TU}^{k}}{\underset{i=1}{\sum }}P_{T}^{T}F_{TU,i}^{k}\right\|}_{F}^{2}\right)=\min_{P_{S},P_{T},P_{TL}}{\left\|N_{S}^{T}F_{S}^{T}P_{S}+N_{TL}^{T}F_{TL}^{T}P_{TL}-N_{TU}^{T}F_{TU}^{T}P_{T}\right\|}_{F}^{2}\text{,} \end{array}$$

where ${\hat{y}}_{TU}$ and *n*_*TU*_ are the pseudolabel vector and the size of the unlabelled target domain *F*_*TU*_, respectively, *F*_*TU*,*i*_^*k*^ and *n*_*TU*_^*k*^ are separately the *i*th tangent feature vector and the size of the unlabelled target domain predicted to be the *k*th class. Let $N_{TU}={\hat{Y}}_{TU}/n_{TU}\text{,}$ in which ${\hat{Y}}_{TU}$ is the one-hot encoding matrix of ${\hat{y}}_{TU}$. For simplicity, *P*_*T*_ is temporally used as the projection matrix of the unlabelled target domain since it relates to the unlabelled target domain.

Finally, we keep and update parameter transfer and regularization since there are abundant tangent feature vectors in the target domain. Then, we want the projection matrix *P*_*T*_ learned in the entire target domain to be similar to the projection matrix *P*_*S*_ learned in the source domain and to be similar to the projection matrix *P*_*TL*_ learned in the labelled target domain. For better generalization performance, we avoid extreme values for these projection matrices. Therefore, we redefine the following constraints:

(27) $$\begin{array}{c} \min_{P_{S},P_{T},P_{TL}}\left({\left\|P_{S}-P_{T}\right\|}_{F}^{2}+{\left\|P_{TL}-P_{T}\right\|}_{F}^{2}+{\left\|P_{T}\right\|}_{F}^{2}\right)\text{.} \end{array}$$

After integrating the regularization terms and the constraints above, the overall loss function of ssMEKT can be formulated as follows:

(28) $$\begin{array}{c} \min_{P_{S},P_{T},P_{TL}}\left({\alpha D}_{L,U}+\beta tr\left(P_{S}^{T}S_{w}^{s}P_{S}\right)+\gamma tr\left(P_{T}^{T}F_{T}LF_{T}^{T}P_{T}\right)+\theta \left({\left\|P_{S}-P_{T}\right\|}_{F}^{2}+{\left\|P_{TL}-P_{T}\right\|}_{F}^{2}+{\left\|P_{T}\right\|}_{F}^{2}\right)\right),subject\;to:P_{S}^{T}S_{b}^{s}P_{S}=I,P_{T}^{T}F_{T}H_{n_{T}}F_{T}^{T}P_{T}=I\text{.} \end{array}$$

Given an overall projection matrix *P* = [*P*_*S*_;*P*_*T*_; *P*_*TL*_](*P* ∈ *ℝ*^3*d*×*p*^), the corresponding Lagrange function can be reformulated as

(29) $$\begin{array}{c} T=tr\left(P^{T}\left(\alpha A+\beta B+\gamma E+\theta G\right)P+\mu \left(I-P^{T}JP\right)\right)\text{,} \end{array}$$

where

(30) $$\begin{array}{c} A=\left[\begin{array}{ccc} {F_{S}N_{S}N}_{S}^{T}F_{S}^{T} & -F_{S}N_{S}N_{TU}^{T}F_{TU}^{T} & F_{S}N_{S}N_{TL}^{T}F_{TL}^{T}\\ {-F}_{TU}N_{TU}N_{S}^{T}F_{S}^{T} & F_{TU}N_{TU}N_{TU}^{T}F_{TU}^{T} & -F_{TU}N_{TU}N_{TL}^{T}F_{TL}^{T}\\ F_{TL}N_{TL}N_{S}^{T}F_{S}^{T} & -F_{TL}N_{TL}N_{TU}^{T}F_{TU}^{T} & F_{TL}N_{TL}N_{TL}^{T}F_{TL}^{T} \end{array}\right],B=\left[\begin{array}{ccc} S_{w}^{s} & 0 & 0\\ 0 & 0 & 0\\ 0 & 0 & 0 \end{array}\right],E=\left[\begin{array}{ccc} 0 & 0 & 0\\ 0 & F_{T}LF_{T}^{T} & 0\\ 0 & 0 & 0 \end{array}\right],G=\left[\begin{array}{ccc} I & -I & 0\\ -I & 3I & -I,\\ 0 & -I & I \end{array}\right]J=\left[\begin{array}{ccc} S_{b}^{s} & 0 & 0\\ 0 & F_{T}H_{n_{T}}F_{T}^{T} & 0\\ 0 & 0 & 0 \end{array}\right]\text{.} \end{array}$$

Then, we can obtain the optimal *P*, *P*_*S*_,*P*_*T*_ and *P*_*TL*_ in the same way as MEKT and sMEKT. Like MEKT, *N*_*TU*_ is updated along with the change of ${\hat{Y}}_{TU}$ at each iteration.

Finally, we choose and average the most relevant projection matrices *P*_*T*_ and *P*_*TL*_ for the projection matrix of unlabelled target domain:

(31) $$\begin{array}{c} P_{TU}=\frac{\left(P_{T}+P_{TL}\right)}{2}\text{.} \end{array}$$

### 3.9. Classification

As depicted in Figure 1, the tangent feature vector sets from different domains can be transformed into new feature sets by different MEKT based algorithms. However, only labelled feature sets are inputted to the supervised sLDA classifier to build a subject-specific model. For MEKT and ssMEKT, the pseudolabels of the new unlabelled target features marked by the model can be used to iteratively update the projection matrices and the model.

Additionally, the goal of LDA is to find an optimal hyperplane that can simultaneously maximize the between-class variances and minimize the within-class variances of the two-class projection data. To cope with high-dimensional data, sLDA uses a shrinkage estimate for the average covariance matrix of each class in the LDA algorithm. More details can be seen in [52].

## 4. Experimental Results

### 4.1. Baseline Algorithms

We compared the following six baseline algorithms with various properties of transfer learning:

1. CSP-LDA is the classical combination of feature extraction and classifier for MI. No source domain is used at all. Only the labelled target domain is used to design the CSP spatial filters and then to train the LDA classifier [33].
2. RA-CCSP-LDA separately performs RA for the labelled source and target domains as in (7), then concatenates them with equal weight to calculate the CSP spatial filters, and finally inputs them into the LDA classifier [38].
3. RA-RCSP-LDA is similar to RA-CCSP-LDA except for the way of generating the spatial filters. RCSP weights the labelled source and target domains using different regularization parameters [35]. To relieve the computational cost and give bigger weight to the labelled target domain, we manually set the two regularization parameters to be 0.1.
4. RA-CCSP-wAR successively executes RA and CCSP before wAR. wAR is also a semisupervised transfer learning algorithm, which performs weighted domain adaptation between the labelled source domain and the entire target domain using SVM as the base classifier [49].
5. RA-RCSP-wAR sequentially performs RA, RCSP, and wAR. For RA-CCSP-wAR and RA-RCSP-wAR, the hyperparameters of wAR were set according to its corresponding publication [49]. Note that three pairs of spatial filters were used for all spatial filtering-based algorithms in our experiments.
6. MEKT-sLDA first performs unsupervised MEKT and then feeds the new labelled features from the source domain into the supervised sLDA classifier [32].

As mentioned above, our proposed sMEKT and ssMEKT also use the sLDA classifier. Moreover, we set the same values for the same weights and parameters in the MEKT based algorithms, such as *α*=1, *β*=0.01, *γ*=0.1, *θ*=20, *μ*=10^−3^,*σ*=1, *K*=10, and *q*=10. Additionally, both MEKT and ssMEKT perform five iterations to update their overall loss functions.

A summary of the six baseline algorithms and the two proposed algorithms is shown in Table 1.

### 4.2. Experimental Design

For each dataset, all trials from each target subject were randomly partitioned into two portions over twenty repetitions. The first portion was the labelled set to train a subject-specific classifier for the supervised and semisupervised algorithms, while the second portion was the unlabelled set to build the classifier for the unsupervised and semisupervised algorithms and to evaluate the effectiveness of different algorithms. All trials from the remaining source subjects were labelled and concatenated into a source domain to be transferred into a target domain. For each target subject, we varied the number of labelled trials from 10 to 50 with a step of 10 to investigate the robustness of all algorithms. For simplicity, denote the trial from the target subject and the trial from the source subject as the target trial and the source trial, respectively.

### 4.3. Classification Accuracy with Few Labelled and/or More Unlabelled Target Trials

The goal of our proposed algorithms is to achieve good classification performance even using few labelled target trials. Thus, we first conducted the experiments with few labelled and/or more unlabelled target trials. For the two MI datasets, ten labelled target trials, with equal number per class, were randomly selected over twenty repetitions. Then, 270 and 190 unlabelled target trials were separately available for dataset 1 and dataset 2. For each target subject, the classification accuracy of the unlabelled target trials was taken as an average of twenty repetitions. Detailed results of the two MI datasets are given in Tables 2 and 3. The bold-faced and italic numbers show the best and second-best classification accuracies, respectively.

Defining BCI illiteracy has been challenging because different researchers use different criteria to distinguish between good and bad subjects. Early work stated that 70% accuracy was effective for binary classification [58, 59]. In Table 2, the nontransfer learning algorithm CSP-LDA reaches or approaches a benchmark accuracy of 70% for subjects al, aw, and ay. Therefore, in our paper, subjects al, aw, and ay are grouped into good subjects, whereas subjects aa and av are grouped into bad ones. The transfer learning algorithms obviously improve the classification performance for bad subjects aa and av. Only MEKT-sLDA and ssMEKT-sLDA obtain satisfactory accuracy for bad subject av. Furthermore, sMEKT-sLDA outperforms the other supervised transfer learning algorithms RA-CCSP-LDA and RA-RCSP-LDA on average when the number of labelled target trials is as low as 10. RA-CCSP-wAR and RA-RCSP-wAR perform slightly higher than their corresponding supervised transfer learning algorithms. Our proposed ssMEKT-sLDA stands out itself among all semisupervised transfer learning algorithms.

In Table 3, CSP-LDA provides the benchmark accuracy of about 70% for subjects e, f, and g, leading to the following categorization in our paper: good subjects e, f, and g; bad subjects a, b, c, and d. The improvement in the classification performance of the transfer learning algorithms is substantial compared to CSP-LDA for bad subjects a, b, c, and d. However, sMEKT-sLDA yields worse classification performance than RA-CCSP-LDA and RA-RCSP-LDA on average. A possible explanation is that more than half of the subjects on dataset 2 perform MI tasks poorly. For sMEKT-sLDA, the target domain can be seriously affected by the bad source domain since the target and source domains are simultaneously adapted to make them close during the minimization of the joint probability distribution shift. However, for RA-CCSP-LDA and RA-RCSP-LDA, both the target and source domains are used to design the spatial filters without directly influencing each other. In addition, RA-CCSP-wAR and RA-RCSP-wAR still perform better than their corresponding supervised transfer learning algorithms.

As shown in Tables 2 and 3, ssMEKT-sLDA exhibits slightly higher classification performance than unsupervised MEKT-sLDA due to the existence of ten labelled target trials. In addition, for the target subjects with 10 labelled samples and 270/190 unlabelled samples, ssMEKT-sLDA shows a 5.27% and 2.69% increase in average accuracy on the two datasets compared to the best semisupervised algorithm RA-RCSP-wAR, respectively.

Then, we performed the paired-sample *t*-tests between the six baseline approaches and our proposed algorithms on the two datasets to further check if the performance differences among all algorithms were significant. The *p*-values are shown in Table 4.

The paired-sample *t*-tests show that the results of our proposed algorithms are statistically higher than those of CSP-LDA (*p* < 0.005). Although our proposed algorithms are based on MEKT-sLDA, their performance differences are very big. In most cases, ssMEKT-sLDA shows its superiority in terms of the *p*-values, especially on dataset 1. The performance differences between sMEKT-sLDA and other transfer learning algorithms are small when sMEKT-sLDA performs well.

### 4.4. Classification Accuracy with Varying Numbers of Labelled and/or Unlabelled Target Trials

**Figure 4:**
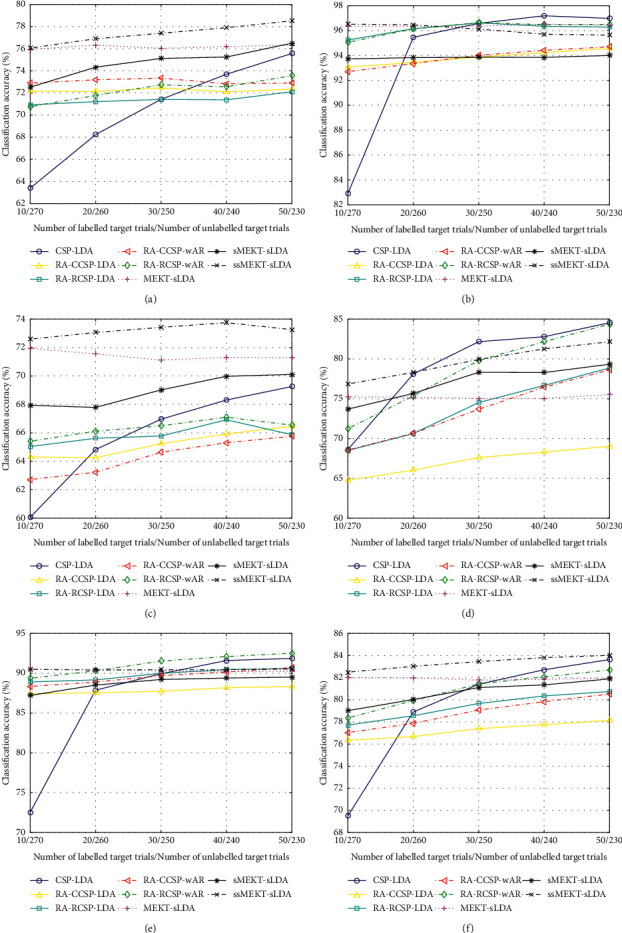
Classification accuracy (%), with varying numbers of labelled and/or unlabelled target trials on dataset 1, for CSP-LDA [33], RA-CCSP-LDA [38], RA-RCSP-LDA [35], RA-CCSP-wAR [49], RA-RCSP-wAR [49], MEKT-sLDA [32], and our proposed algorithms. (a) Subject aa. (b) Subject al. (c) Subject av. (d) Subject aw. (e) Subject ay. (f) All subjects.

**Figure 5:**
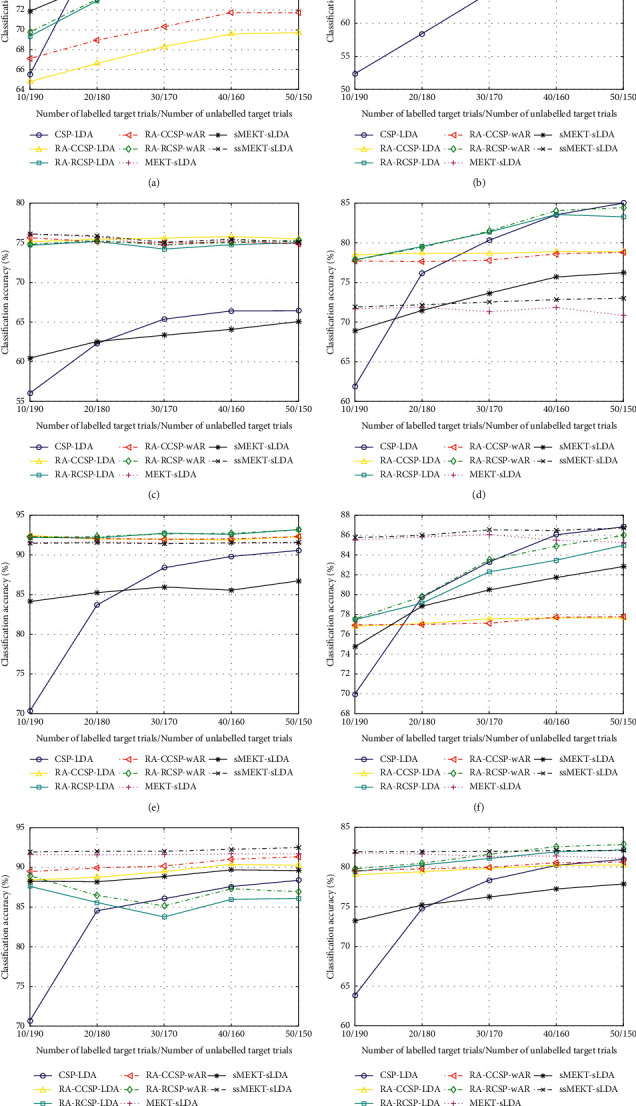
Classification accuracy (%), with varying numbers of labelled and/or unlabelled target trials on dataset 2, for CSP-LDA [33], RA-CCSP-LDA [38], RA-RCSP-LDA [35], RA-CCSP-wAR [49], RA-RCSP-wAR [49], MEKT-sLDA [32], and our proposed algorithms. (a) Subject a. (b) Subject b. (c) Subject c. (d) Subject d. (e) Subject e. (f) Subject f. (g) Subject g. (h) All subjects.

To show the effect of the size of the target domain, with varying numbers of labelled and/or unlabelled target trials, the classification accuracies of different subjects from different datasets and their means are shown in Figures 4 and 5.

As depicted in Figures 4(b), 4(d), and 4(e), for good subjects al, aw, and ay, CSP-LDA shows better classification performance than the supervised and semisupervised transfer learning algorithms with the increase of the labelled target trials. As shown in Table 2, these subjects perform well even using few labelled target trials. Thus, as their labelled target trials increase, their increasing between-class discriminability can gradually reduce their dependence on transfer learning. As illustrated in Figures 4(a) and 4(c), for bad subjects aa and av, most transfer learning algorithms outperform CSP-LDA in most cases. It is necessary for them to transfer the source labelled trials due to their poor between-class discriminability. In Figures 4(a), 4(b), 4(c), and 4(e), the average performance of sMEKT-sLDA is higher than that of RA-RCSP-LDA for bad subjects aa and av, while RA-RCSP-LDA performs better than sMEKT-sLDA on average for good subjects al and ay. The reason is that much more good source subjects are available for bad target subjects aa and av, compared to good target subjects al and ay. Bad target subjects aa and av benefit from the domain adaptation used in sMEKT-sLDA, while good target subjects al and ay benefit from their bigger weights used in RA-RCSP-LDA. As shown in Figure 4(f), for all subjects, the classification performance of MEKT-sLDA decreases due to the reduction of unlabelled target trials. On average, ssMEKT-sLDA shows its compelling validity.

As shown in Figures 5(e)–5(g), for good subjects e, f, and g, due to their good between-class discriminability, as the labelled target trials increase, the classification performance of CSP-LDA is close to that of the transfer learning algorithms. As depicted in Figures 5(b) and 5(c), for bad subjects b and c, due to their poor between-class discriminability, the transfer learning algorithms maintain obvious advantages over CSP-LDA with the increasing labelled target trials. As illustrated in Figures 5(a)–5(h), on average, sMEKT-sLDA performs worse than most supervised transfer learning algorithms since a poor source domain might affect the discriminability of the target domain during domain adaptation. For subjects a, d, e, and f, RA-RCSP-wAR achieves higher results than RA-CCSP-wAR in most cases. All semisupervised transfer learning algorithms reach better performance than their supervised or unsupervised counterparts. Moreover, ssMEKT-sLDA shows its superiority for four out of seven subjects.

### 4.5. Computational Cost

All experimental results were obtained from our operating platform:

1. Hardware: processor: Intel i5 CPU@1.60 GHz; RAM: 8 GB.
2. Software: Windows 10 Home Edition; Matlab 2015a.

The computation times of the different algorithms on two datasets using 10 labelled and/or 270/190 unlabelled target trials are shown in Table 5. The best performance was highlighted in bold.

As shown in Table 5, CSP-LDA spends the shortest computation time among all algorithms. The computational cost of sMEKT-sLDA is slightly higher than that of RA-RCSP-LDA due to higher-dimensional features. ssMEKT-sLDA requires more time than other MEKT based algorithms because of more available trials. However, for all semisupervised transfer learning algorithms, even with higher-dimensional features, ssMEKT-sLDA runs faster than RA-CCSP-wAR and RA-RCSP-wAR on average. The possible reason is that wAR works more complicatedly than ssMEKT.

## 5. Discussion

In this section, we discuss the experimental results from various aspects.

### 5.1. Effectiveness of Riemannian Alignment and Transfer Learning

In our experiments, all algorithms perform RA and transfer learning, except CSP-LDA. They first execute RA for different domains in an unsupervised way, which can not only make different domains comparable, but also overcome the impact of limited labelled target trials. For all spatial filtering-based transfer learning algorithms, such as RA-CCSP-LDA, RA-RCSP-LDA, RA-CCSP-wAR, and RA-RCSP-wAR, the EEG trials are whitened by performing RA. Likewise, for all MEKT based transfer learning algorithms, the tangent feature vectors from different subjects are close to each other due to RA. Thus, RA can shorten the differences between domains, which is beneficial to successive transfer learning. As mentioned above, different transfer learning algorithms combine different domains in different ways. In Tables 2 and 3, even using few labelled and/or more unlabelled target trials, with the help of abundant labelled source trials, the supervised, unsupervised, and semisupervised transfer learning algorithms demonstrate better average classification accuracies than CSP-LDA. It is implied that both RA and transfer learning contribute to the good classification performance.

However, as shown in Figures 4 and 5, for good target subjects al, aw, ay, e, f, and g, with the increase of their labelled trials, their between-class discriminability can increase; thus the classification accuracies of CSP-LDA are gradually close to, or even higher than those of transfer learning algorithms. In contrast, for bad target subjects aa, av, b, and c, the transfer learning algorithms always outperform CSP-LDA as the labelled target trials increase. Therefore, it is implied that the number of labelled target trials and the extent of between-class discriminability of the source subjects greatly affect the performance of transfer learning algorithms.

### 5.2. Differences between Spatial Filtering and Manifold Embedded Knowledge Transfer

The CSP features used in all spatial filtering-based transfer learning algorithms have lower dimensions than the transformed tangent space vectors used in all MEKT based transfer learning algorithms. To keep or highlight the importance of the target domain, for CCSP based algorithms, the weight of the target domain is the same as that of the source domain, and for RCSP based algorithms, the weight of the target domain is higher than that of the source domain. Consequently, the source and target domains independently play their roles in spatial filtering-based algorithms. However, to further reduce the differences between the source and target domains, all MEKT based transfer learning algorithms pay great attention to the joint probability distribution shift minimization between domains. Therefore, the target domain is easily affected by the source domain during the minimization. The degree of positive transfer depends on the performance of the source domain.

As shown in Tables 2–4, most MEKT based transfer learning algorithms show better performance than the spatial filtering-based ones. However, for dataset 2, the average classification accuracy of sMEKT-sLDA is inferior to that of spatial filtering-based transfer learning algorithms, but still superior to that of CSP-LDA. The possible reason is that only three out of seven subjects perform MI tasks well on dataset 2. Thus, the poor source domain affects the positive transfer of sMEKT-sLDA. Thus, our supervised transfer learning algorithm sMEKT-sLDA should identify the most suitable source subjects instead of all available source subjects. In addition, due to the existence of unlabelled target domain, MEKT-sLDA and ssMEKT-sLDA reduce the negative impact of the poor source domain. As given in Table 5, in terms of computation time, all supervised transfer learning algorithms run efficiently. Furthermore, ssMEKT-sLDA takes shorter time than RA-CCSP-wAR and RA-RCSP-wAR. Overall, our proposed MEKT based algorithms provide comparably good performance with efficient running time.

### 5.3. Impact of Labelled and/or Unlabelled Target Trials

To investigate the roles of labelled and unlabelled target trials, all algorithms can be divided into supervised, unsupervised, and semisupervised algorithms. As shown in Tables 2 and 3, with abundant unlabelled target trials, MEKT-sLDA outperforms spatial filtering-based algorithms and sMEKT-sLDA on average. Thus, a large unlabelled target domain is beneficial for classification due to its embedded geometric structure. Moreover, with the help of a few labelled target trials, the performance of ssMEKT-sLDA is slightly better than that of MEKT. As illustrated in Figures 4 and 5, with increasing number of the labelled target trials and decreasing number of the unlabelled target trials, the performance improvement of ssMEKT over MEKT increases. As depicted in Figures 4(f) and 5(h), the curve of CSP-LDA grows fast with the increase of the labelled target trials. It is implied that the labelled target trials are crucial for the classification performance. Since the labelled and unlabelled target trials are less than the labelled source trials, the performance improvements of supervised and semisupervised transfer learning are not apparent as the labelled target trials increase.

Additionally, as shown in Figures 4 and 5, for good target subjects al, ay, e, f, and g, their classification accuracies of ssMEKT-sLDA are always similar to those of MEKT-sLDA. The possible reason is that both the unlabelled and labelled target trials of good target subjects can provide important information. However, for bad target subjects aa, av, a, and d, the differences between ssMEKT-sLDA and MEKT-sLDA are comparably obvious. The possible explanation is that the labelled target trials of bad target subjects can provide more valuable information than their unlabelled target trials. Overall, labelled target trials play an important role in the classification. The unlabelled target trials are also beneficial for classification, especially for good target subjects.

It is noted that our proposed ssMEKT-sLDA does not utilize the unlabelled target trials to train the classifier, resulting in the limited performance improvement.

## 6. Conclusion

To shorten the calibration time of the target subject, we propose a supervised MEKT algorithm (sMEKT) and a semisupervised MEKT algorithm (ssMEKT) in MI-based BCI. Both are combined with the sLDA classifier. Due to high intersubject variability, it is better to build a subject-specific classifier rather than a generic classifier. Both sMEKT and ssMEKT transfer abundant labelled samples from multiple source subjects for a specific target subject. First, they perform RA in an unsupervised way to preliminarily reduce the differences among different subjects. Then, they convert the aligned covariance matrices from different subjects into the corresponding tangent feature vectors for classification in the Euclidean space. Finally, to further cope with variations among different subjects, sMEKT performs domain adaptation between the labelled source and target domains, whereas ssMEKT performs domain adaptation between the labelled source domain and the entire target domain. During adaptation, both sMEKT and ssMEKT not only minimize the joint probability distribution shift among different domains, but also maintain the source domain discriminability as much as possible. Moreover, ssMEKT keeps the entire target domain locality to utilize its geometric structure. In addition, ssMEKT performs the parameter transfer and regularization, to make the projection matrices of different domains closer. For the target subjects with 10 labelled samples and 270/190 unlabelled samples, ssMEKT stands out itself with average accuracies of 82.50% and 81.94% on the dataset 1 and dataset 2, respectively, and sMEKT can also obtain higher classification performance (79.02%) than other spatial filtering-based transfer learning algorithms on dataset 1. Therefore, the experimental results show that our proposed algorithms can reduce the need of labelled target trials. In the future, we will not only choose the most beneficial source subjects for the target subjects, but also make use of the unlabelled target samples in the classification module. In addition, our proposed ssMEKT is designed offline since the unlabelled samples from the target subject are obtained a priori, instead of on-the-fly. Future work will be dedicated to updating ssMEKT in real-time BCI applications, where domain adaptation is performed between the labelled source domain and the increasing target domain.

## Figures and Tables

**Table 1:** A summary of the eight algorithms with various properties.

	Spatial filtering	RA	Transfer learning	Supervised	Semisupervised	Unsupervised
CSP-LDA [33]	√	—	—	√	—	—
RA-CCSP-LDA [38]	√	√	√	√	—	—
RA-RCSP-LDA [35]	√	√	√	√	—	—
RA-CCSP-wAR [49]	√	√	√	—	√	—
RA-RCSP-wAR [49]	√	√	√	—	√	−
MEKT-sLDA [32]	—	√	√	—	—	√
sMEKT-sLDA	—	√	√	√	—	—
ssMEKT-sLDA	—	√	√	—	√	—

**Table 2:** Classification accuracy (%) with 10 labelled target trials and/or 270 unlabelled target trials on dataset 1, for CSP-LDA [33], RA-CCSP-LDA [38], RA-RCSP-LDA [35], RA-CCSP-wAR [49], RA-RCSP-wAR [49], MEKT-sLDA [32], and our proposed algorithms.

	aa	al	av	aw	ay	Mean ± std.
CSP-LDA	63.39	82.89	60.06	68.61	72.54	69.50 ± 8.89
RA-CCSP-LDA	72.17	93.06	64.31	64.76	87.41	76.34 ± 13.21
RA-RCSP-LDA	70.93	95.24	65.04	68.50	88.91	77.72 ± 13.45
RA-CCSP-wAR	72.89	92.70	62.72	68.56	88.35	77.04 ± 12.92
RA-RCSP-wAR	70.76	95.06	65.41	71.24	89.37	78.37 ± 13.00
MEKT-sLDA	*75.94*	*96.35*	*71.94*	*75.26*	*90.48*	*82.00* ± 10.74
sMEKT-sLDA	72.52	93.72	67.94	73.69	87.22	79.02 ± 10.92
ssMEKT-sLDA	**76.06**	**96.52**	**72.59**	**76.85**	**90.50**	**82.50** ± 10.39

**Table 3:** Classification accuracy (%) with 10 labelled target trials and/or 190 unlabelled target trials on dataset 2, for CSP-LDA [33], RA-CCSP-LDA [38], RA-RCSP-LDA [35], RA-CCSP-wAR [49], RA-RCSP-wAR [49], MEKT-sLDA [32], and our proposed algorithms.

	a	b	c	d	e	f	g	Mean ± std.
CSP-LDA	65.50	52.37	56.05	61.92	70.34	69.97	70.68	63.83 ± 7.36
RA-CCSP-LDA	64.79	77.03	75.13	**78.50**	**92.45**	76.82	88.42	79.02 ± 9.09
RA-RCSP-LDA	69.37	76.87	74.68	*77.84*	92.21	77.47	87.61	79.44 ± 7.82
RA-CCSP-wAR	67.11	**77.58**	75.63	77.71	*92.32*	76.95	89.47	79.54 ± 8.62
RA-RCSP-wAR	69.79	*77.42*	74.79	*77.84*	92.24	77.53	88.95	79.79 ± 7.94
MEKT-sLDA	*83.24*	72.63	*76.08*	71.71	91.55	*85.50*	*91.61*	*81.76* ± 8.42
sMEKT-sLDA	71.89	64.16	60.47	68.89	84.13	74.74	88.26	73.22 ± 10.11
ssMEKT-sLDA	**83.76**	72.66	**76.11**	71.92	91.47	**85.74**	**91.95**	**81.94** ± 8.46

**Table 4:** The *p*-values in paired-sample *t*-tests on the two datasets between our proposed algorithms and CSP-LDA [33], RA-CCSP-LDA [38], RA-RCSP-LDA [35], RA-CCSP-wAR [49], RA-RCSP-wAR [49], and MEKT-sLDA [32].

	Dataset 1		Dataset 2	
	sMEKT-sLDA	ssMEKT-sLDA	sMEKT-sLDA	ssMEKT-sLDA
CSP-LDA	0.0040	0.0011	0.0027	0.00002
RA-CCSP-LDA	0.1903	0.0246	0.0954	0.4091
RA-RCSP-LDA	0.3813	0.0310	0.0444	0.3883
RA-CCSP-wAR	0.2179	0.0230	0.0573	0.4536
RA-RCSP-wAR	0.5493	0.0264	0.0325	0.4513
MEKT-sLDA	0.0020	0.1546	0.0025	0.0570

**Table 5:** Computation times (seconds) of CSP-LDA [33], RA-CCSP-LDA [38], RA-RCSP-LDA [35], RA-CCSP-wAR [49], RA-RCSP-wAR [49], MEKT-sLDA [32], and our proposed algorithms on two datasets.

	Dataset 1	Dataset 2	Mean ± std.
CSP-LDA	**0.16**	**0.03**	**0.10** ± 0.09
RA-CCSP-LDA	1.36	0.29	0.83 ± 0.76
RA-RCSP-LDA	0.28	0.31	0.30 ± 0.02
RA-CCSP-wAR	6.56	4.50	5.53 ± 1.46
RA-RCSP-wAR	5.42	4.57	5.00 ± 0.60
MEKT-sLDA	1.26	2.60	1.93 ± 0.95
sMEKT-sLDA	0.45	0.50	0.48 ± 0.04
ssMEKT-sLDA	2.47	4.74	3.61 ± 1.61

## Data Availability

In this study, the authors used two publicly available MI datasets for analysis: (1) dataset IVa, BCI competition III [50], which has been deposited on the website https://www.bbci.de/competition/iii/#data_set_iva; (2) dataset 1, BCI competition IV [51], which is available on the website http://www.bbci.de/competition/iv/desc_1.html.
